# Stem cells in Osteoporosis: From Biology to New Therapeutic Approaches

**DOI:** 10.1155/2019/1730978

**Published:** 2019-06-02

**Authors:** Vasilis Paspaliaris, George Kolios

**Affiliations:** ^1^Tithon Biotech Inc., San Diego, CA 92127, USA; ^2^Lab of Pharmacology, Faculty of Medicine, Democritus University of Thrace, Dragana, Alexandroupolis 68100, Greece

## Abstract

Osteoporosis is a systemic disease that affects the skeleton, causing reduction of bone density and mass, resulting in destruction of bone microstructure and increased risk of bone fractures. Since osteoporosis is a disease affecting the elderly and the aging of the world's population is constantly increasing, it is expected that the incidence of osteoporosis and its financial burden on the insurance systems will increase continuously and there is a need for more understanding this condition in order to prevent and/or treat it. At present, available drug therapy for osteoporosis primarily targets the inhibition of bone resorption and agents that promote bone mineralization, designed to slow disease progression. Safe and predictable pharmaceutical means to increase bone formation have been elusive. Stem cell therapy of osteoporosis, as a therapeutic strategy, offers the promise of an increase in osteoblast differentiation and thus reversing the shift towards bone resorption in osteoporosis. This review is focused on the current views regarding the implication of the stem cells in the cellular and physiologic mechanisms of osteoporosis and discusses data obtained from stem cell-based therapies of osteoporosis in experimental animal models and the possibility of their future application in clinical trials.

## 1. Introduction

Osteoporosis is the most common systemic disease of the skeleton with the main feature of the uninterrupted disturbance of bone microarchitecture, resulting, particularly high in the elderly, in an increased risk of bone fractures [1, 2]. Osteoporosis is endemic in developed countries with about 50% of women and 20% of men, over the fifties, being affected in the UK (National Osteoporosis Society, UK) and up to 50 million individuals meeting the criteria of the World Health Organization (WHO) for osteoporosis in industrialized countries in North America, Europe, Japan, and Australia [3]. According to the WHO criteria, the diagnosis of osteoporosis is confirmed when the values of bone mineral density (BMD) are equal to or less than 2.5 standard deviations below the normal, and it is expressed as a *T* − score≤−2.5, with a normal *T* − score being equal of zero [4]. Despite the current pharmaceutical intervention available, bone fractures are still very common in patients with osteoporosis and the associated medical costs are relatively high. In 2005 in the United States of America, the annual cost for the management of patients with fractures from osteoporosis reached 19 billion dollars. Given the increase in life expectancy in the coming years, it is estimated that the cost in 2025 will be higher than $25 billion [5].

The pathophysiological mechanism in osteoporosis is a disturbance of bone metabolism, by several risk factors that cause a dysregulation between bone resorption and new bone formation and disruption of the well-orchestrated procedure of osteogenesis and bone remodeling [6]. Optimal bone metabolism is a dynamic mechanism throughout life where a constant process between the balance of proteolytic bone digestive activity of osteoclasts and the bone-secreting function of osteoblasts takes place [7]. The physiological bone remodeling process that regenerates the entire skeleton has been calculated to take place every 10 years [8]. Furthermore, this bone remodeling also controls the reshaping of the bone following injuries such as major fractures or stress-induced microfractures [9]. When this bone remodeling is imbalanced, where bone formation is decreased and/or bone resorption is increased, osteoporosis occurs. The recruitment and activation of specialized bone cells and their progenitor cells at the site of the remodeling are orchestrated by multiple signal and receptor activation pathways, which is quite complicated and not fully elucidated. These pathways are recently targets for the development of new therapies, based on the regenerative medicine, for the management of osteoporosis [10]. The main two specialized cells that have been mostly studied in bone remodeling are the osteoclast (bone resorption) and osteoblast (bone formation). These two cell types were found to have an interdependent relationship via a variety of mechanisms [11, 12]. Osteoporosis is mediated by several factors that induce both the onset of the disease and its maintenance, such as hormonal, nutritional, and behavioral factors, as well as a genetic predisposition [7, 13]. Bone mass reaches its maximum development within the first decade of adulthood and maintains it for the next years, but with increasing age, both women and men lose bone, with aging having a paramount influence on the onset and the progression of osteoporosis [14]. Osteoporosis is quite clearly more aggressive in postmenopausal women, with a dramatic increase of the rate of bone turnover and a continuous bone loss due to estrogen deficiency [15, 16]. Estrogen deficiency and glucocorticoids represent the most common cause of osteoporosis [17].

During the last decades, stem cells have gained considerable attention, both for their broad use in basic research and for their potential capabilities in the regenerative medicine to develop therapeutic strategies for a large number of pathophysiological disorders [18]. Stem cell therapies have been investigated in almost every degenerative disorder [19–22], and promising results from preclinical studies and clinical trials in several diseases have led to the development of new therapeutic strategies [23, 24]. This review focuses on the field of regenerative medicine that is attempting to develop new stem cell therapeutic strategies for osteoporosis to prevent and repair bone loss and to reduce the susceptibility of fractures in osteoporotic patients [25].

## 2. Overview of Current Therapies

The current therapeutic management of osteoporosis is based on a range of pharmacological agents that inhibit bone resorption or anabolic drugs that induce bone formation [26]. Studies show that the antiabsorption drugs do not restore the loss of bone mass but inhibit its further absorption and bone degradation. Bisphosphonates, which are nonhydrolysable pyrophosphate analogs, inhibit the absorption of bone mass by disrupting the function of osteoclasts and affecting their survival via a disorder of the subcellular localization and the normal function of certain signaling proteins which leads to accumulation of toxic nucleotide metabolites [27]. Although bisphosphonates are the most common treatment of osteoporosis, generally well tolerated and reduce the risk of osteoporotic-related fractures, a number of serious adverse events have been observed to occur [28], such as osteonecrosis of the jaw, gastrointestinal upsets, renal complications, and atypical femoral fractures [29–33]. Another agent that reduces bone loss and minimizes the risk of bone fracture is calcitonin, which is a hormone produced and secreted by the C cells of the thyroid gland. However, the use of calcitonin in long-term treatments has been implicated in an increased risk of malignancies, such as prostate cancer, and has also been found to be less effective in the increase of bone mass or the reduction of bone turnover when administered to postmenopausal women [34–36]. Recently, the European Medicines Agency (EMA) recommended that the benefits from calcitonin did not outweigh their risks in the treatment of osteoporosis, and thus, calcitonin is not typically described or developed as a treatment for osteoporosis [36, 37]. Selective estrogen receptor modulators (SERMs), a class of drugs that bind the estrogen receptor acting as either antagonists or agonists, are used in the treatment of osteoporosis. These factors appear to reduce the risk of osteoporotic bone fractures due to the reduction of the bone loss. Raloxifene and its chemical cousin arzoxifene are therapeutically administered for the prevention of fractures to postmenopausal women suffering from osteoporosis [38, 39]. However, a large randomized study has associated the administration of raloxifene to women with cardiovascular disease, after menopause, with an increased risk of thromboembolic events and strokes [40]. Data suggest that both raloxifene and arzoxifene reduce vertebral fracture risk by improving BMD and improve osteoporosis in postmenopausal women; however, careful consideration is required when prescribing SERMs, mainly to patients with previous history of venous thromboembolism [41, 42].

Parathyroid hormone (PTH) is one of the two essential hormones that regulate calcium and phosphate homeostasis in the bones, it is an 84-amino acid polypeptide, and it is secreted by the parathyroid glands. Administration of PTH in an intermittent manner and at low doses shows an anabolic effect inducing bone remodeling [43]. Teriparatide is a recombinant form of PTH that consists of the bioactive part of the hormone, the first 34 amino acids of the N-terminus. This agent is used for osteoporosis treatment and, in contrast to drugs that act as inhibitors of bone loss, mentioned above, induces bone formation by targeting recruitment of new osteoblasts (osteoblastogenesis) and osteoblast survival [44]. The use of teriparatide, however, is limited for up to 2 years [45], and upon termination of treatment, the patient must continue with antiresorptive drugs as discontinuation of the treatment with PTH rapidly results in a decrease of the BMD [46]. Despite the limitation, the use of teriparatide for the treatment of osteoporosis seems to increase the rate of healing of bone fractures resulting in improved functionality in osteoporotic patients [44]. Calcium sensing receptor (CaSR), a G-protein-coupled receptor, is expressed on the surface of parathyroid cells, and it is activated by millimolar concentrations of extracellular Ca2+, which primarily regulates the secretion of PTH. Calcimimetics are a class of drugs that activate this receptor and inhibit the secretion of PTH, while calcilytics are CaSR antagonists and they stimulate the secretion of PTH. Accumulating evidence indicates that this receptor is implicated in the regulation of bone metabolism [47], and it has been suggested that this receptor could be a potential target in drug development for the management of bone metabolism disorders [48, 49]. Activation of CaSR, by a calcimimetic compound, using amniotic fluid-derived human mesenchymal stem cells, demonstrated a significant enhancement of their osteogenic differentiation after exposure in the osteogenic environment [50]. Calcilytics were proposed for the management of osteoporotic patients [49], because these compounds have shown an anabolic efficacy, in OVX rat models of osteoporosis [51, 52]. However, the results from phase II clinical trials in postmenopausal osteoporotic women were disappointing [53, 54] and their clinical development was stopped, due to the lack of clinical efficacy [55]. Despite the experimental evidence that compounds that act on the PTH receptor are involved in bone regeneration, there is no clinical evidence to support their efficacy in the treatment of osteoporosis. Recently, a metanalysis has demonstrated that combination therapy with PTH analogs and antiresorptive agents was more successful than monotherapy in osteoporotic patients [56]. However, it is based in a small number of patients and large-scale studies are necessary to evaluate these results.

Strontium is a chemical element with similar chemical properties to those of calcium, its vertical neighbor in the periodic table. This similarity gives the strontium the ability to substitute calcium in the bone tissue without disturbing the physiology of bone metabolism [57]. Strontium ranelate, a strontium salt of ranelic acid, is used in the treatment of osteoporosis, as it has the capacity to reduce the risk of bone fractures, including vertebral compression fractures and to increase BMD [58]. This therapeutic effect seems to be the result of dissociation of bone resorption from the bone formation, leading to the increase of functional osteoblasts and simultaneous decrease of osteoclasts [59]. Strontium ranelate is an alternative treatment, with a positive benefit-harm relationship, for osteoporosis, but consideration should be given to the contraindications, the revised indications, and the possible cardiovascular risk [58, 60]. Skin rashes, myocardial infarction, and embolic and thrombotic venous events have been reported as side effects from the use of strontium ranelate in the treatment of osteoporosis [58].

The introduction of the biological therapies in the last decades to clinical practice has also given new impetus to new therapeutic approaches and the development of new agents in the management of osteoporosis. Denosumab, an IgG2 human monoclonal antibody, is a biological agent for the treatment of osteoporosis. This agent exhibits high specificity and affinity for the receptor activator of nuclear factor kappa-B ligand (RANKL) [61]. It exerts antiresorptive properties by inhibiting the RANKL to interact with its receptor on the surface of osteoclasts and by blocking formation, function, and survival of osteoclasts and by reducing bone resorption [62]. Denosumab is considered as a relatively safe treatment of osteoporosis [63]. Monoclonal antibodies directed to inhibiting sclerostin and cathepsin K inhibitors have been generated for the treatment of osteoporosis. Three humanized neutralizing antibodies to sclerostin, a glycoprotein secreted by osteocytes with a negative effect on the formation of bone tissue, are currently in clinical trials, under development, with preclinical and clinical evidence supporting their use in osteoporosis [64]. A number of compounds that inhibit cathepsin k have also been used to treat osteoporosis. However, most of them caused adverse reactions and drug interactions, resulting in withdrawal [65]. Odanacatib, a cathepsin k inhibitor, which directly inhibits bone resorption and maintains bone formation, was developed up to phase III clinical trials in osteoporotic postmenopausal women [66] but has been discontinued due to the increased risk of stroke and major adverse cardiovascular events [67].

The limited efficacy and the undesirable side effects of both the existing antiresorptive and the anabolic drugs, combined with the failed efforts of newly developed drugs in clinical trials on new therapeutic agents, increase the importance of developing new therapeutic strategies that could promote bone regeneration in individuals with osteoporosis. Recently, extensive research has focused on bone regeneration through biomaterial scaffolds, small osteoinductive molecules, and stem cells of various origin, for the therapy of orthopedic-related diseases, mainly osteoporosis [68, 69]. Various biomaterials, natural or synthetic, have been examined for their use as grafts for the repair of bone fractures in osteoporotic patients [70]. Thus, scaffolds from ceramics or from bioderived materials, such as the extracellular matrix, and synthetic scaffolds from polymers or fibers have been designed and used to mimic native bone and provide the necessary biochemical and mechanical support for stem cells to adhere, proliferate, and differentiate for ingrowth of new bone [71–75]. Further research is needed to examine which types of scaffolds can reconstitute native tissue biological properties and what stem cell combinations could induce and maintain a functional and stable osteoblast population to generate new bone tissue [68]. Osteoinductive molecules, such as growth factors, peptides, and small molecules, with osteogenic properties have also been used in tissue regeneration and tissue engineering [76]. These molecules can induce both angiogenesis and osteogenesis simultaneously, and a number of small osteoinductive molecules alone or combined with other osteogenic techniques are under investigation for the treatment of osteoporosis [77–80]. However, stem cells have gained considerable experimental and clinical attention for the development of cell-based therapies due to their promising contribution to regenerative medicine to date.

## 3. Stem Cells in the Pathogenetic Mechanism of Osteoporosis

### 3.1. Stem Cells in Bone Regeneration and Pathophysiology of Osteoporosis

Osteogenesis and bone remodeling are complex processes that involve multiple mechanisms and interactions between distinct cell populations, not only the osteoblastic and osteoclastic cell lineages [81]. An interplay between progenitor stem cells that maintain the bone cell repertoire and hematopoietic and tissue immune cells also occurs, via the secretion of local cytokines and growth factors [82–84] and the activation of transcription factors [85, 86]. Osteoporosis is a metabolic disorder, which implicates not only the osteoblast and osteoclast imbalance but also other functions, such as bone-vessel coupling and bone-adipocyte coupling [87]. Despite the complex nature of the pathogenesis of osteoporosis, osteoblast generation is the most important mechanism of its pathogenetic process since osteoid secretion by osteoblasts is the main procedure for producing bone tissue. Accumulating evidence has indicated an important role of a stem cell population, derived from bone marrow, in bone maintenance and remodeling. Recently, it has been identified and a population of self-renewing and multipotent human skeletal stem cells (SSCs) that generates progenitors of bone, cartilage, and stroma, but not fat has been reported [88]. These multipotent SSCs reside in the postnatal bone marrow and are implicated in skeletal physiology [89]. The term mesenchymal stem cells (MSCs) has been used for years in literature to characterize a multipotent stromal cell population that can differentiate into a variety of cell types, including osteoblasts (bone cells) and chondrocytes (cartilage cells), and most articles use this term when they refer to multipotent skeletal stem cells [90–92]. The bone tissue homeostasis, under physiological conditions, is maintained due to the osteogenetic and adipogenetic abilities of the MSCs [93]. Various factors such as menopause and aging or ovariectomy could disrupt this homeostatic ability of the MSCs that would lead to the accumulation of a large number of bone marrow adipocytes resulting in bone mass loss and osteoporosis [94]. The proliferative response and the osteogenic differentiation of MSCs have been found significantly affected in osteoporosis [95]. The differentiation of MSCs into osteoblasts promotes osteogenesis; however, a balance between their differentiation into osteoblasts and adipocytes is necessary to maintain the balance between bone and adipose tissues. If the number of adipocytes increases and that of osteoblasts decreases, osteoporosis could result [96]. Understanding the regulatory factors of the osteogenic and adipogenic abilities of MSC is very important in the study of the underlying mechanisms governing osteoporosis. The differentiation of MSCs involves the transformation from osteogenic cells to preosteoblasts and finally into osteoblasts. Following differentiation, the osteoblasts proliferate, maturate, and secrete extracellular matrix proteins to induce bone matrix mineralization [97]. The sequential process of MSC differentiation to bone-forming osteoblasts and how it is affected by factors such as hormones, cytokines, drugs, and physiotherapy are very important for understanding normal growth and maintenance of bone tissue [98]. Dysfunction at any stage of this sequential procedure leads to a disorder of the bone metabolism and osteoporosis, as a result of an imbalance between bone formation and resorption [93]. Thus, the therapeutic strategies for osteoporosis must target the improvement of MSC differentiation into osteoblasts and the osteoblast proliferation.

In healthy individuals, these MSC-derived osteoblast progenitors occur at a very low rate in the bone marrow [99]. In elderly people, the number of osteoblast progenitor MSCs is further decreased, leading to reduction in bone formation and osteoporosis [100]. Apart from the progenitor number reduction, age-related osteoporosis is characterized by diminished ability of MSCs to proliferate and differentiate giving osteoblasts capable of forming bone [101]. The capacity of MSCs from osteoporosis patients to differentiate into osteoblasts was found to be lower than that from healthy people, as they are found to have a lower growth rate than control cells and to exhibit a deficient ability to differentiate into the osteogenic lineage [102]. Therefore, bone formation and remodeling are dependent not only on the appropriate number but also on the function of the osteoblast progenitor MSCs [103]. MSCs from postmenopausal women with osteoporosis were found to have less sensitivity to insulin-like growth factor and a weaker ability to differentiate into osteoblasts, compared to normal controls, while postmenopausal women with osteoporosis were found to express a differential responsiveness of MSCs to leptin, resulting to a variability of the MSC osteogenic potential [104].

Many studies have shown that two factors, estrogen and glucocorticoids, have an important regulatory effect on the pathophysiology of osteoporosis affecting the differentiation of MSCs. Estrogen receptor, a type of nuclear hormone receptor, is expressed in the osteoblasts, and it has an extremely important role in maintaining the equilibrium between bone resorption and formation [105]. Various studies have confirmed that estrogen, in certain concentrations, can induce MSC proliferation and osteogenic differentiation [106]. Another study demonstrated that estrogen, in parallel with the induction of osteogenic differentiation of the MSCs, inhibited adipogenic differentiation [107]. Estradiol was found to promote MSC-derived osteoblast proliferation and maturation and the expression and secretion of bone morphogenetic protein 2 (BMP2) [108]. These studies suggest that hormone replacement therapy could have a therapeutic effect on osteoporosis, but long-term administration of estrogen in postmenopausal women has been found responsible for increased incidences of coronary heart disease, stroke, and breast cancer [109]. Recently, a role of IL-17 has been suggested in the estrogen-deficient osteoporosis. IL-17 was found to promote osteoclastogenesis, in a mice model of ovariectomy- (OVX-) induced osteoporosis, by stimulating RANKL expression by osteoblasts via the IL-17RA SEFIR/TILL. Act1mRNA expression that is augmented in estrogen deficiency increases the RANKL expression and causes bone resorption via an IL-17RA interaction [110]. Another study demonstrated that estrogen deficiency increases IL-17 production, modulating differentiation of Th17 cells, and results to production of osteoclastogenic mediators, including IL-6, TNF-*α*, and RANKL from osteoblasts and bone loss. Blocking of IL-17 function, with a neutralizing monoclonal antibody, was found to prevent bone resorption in an OVX mouse experimental model [111].

Transplantation of MSCs in systemic lupus erythematosus mice was found to ameliorate secondary osteoporosis, rendering IL-17 production in bone marrow and improving the function of MSCs. This resulted in a balance between osteoblasts and osteoclasts and maintenance of a positive bone metabolism [112]. In addition to the implication of estrogens in the pathophysiology of postmenopausal osteoporosis, these results support the evidence that osteoporosis is associated with disorders of innate immunity.

Glucocorticoids also have a regulatory effect on the differentiation of MSCs and osteoporosis. Long-term use or/and high-doses of glucocorticoids have been found to induce osteoporosis via differentiation of MSCs to adipocytes against the differentiation to osteoblasts [113]. It was also reported that high dosage of dexamethasone could lead to apoptosis of bone marrow MSCs, which would result to osteoporosis [114]. Ginsenoside, a pharmacologically active compound found in ginseng, was demonstrated to inhibit the high dosage of dexamethasone-induced apoptosis in primary murine bone marrow MSCs [115]. Thus, when patients are treated with corticosteroids, the dose, the duration of the treatment, and the postmenopausal status should be taken into account [116]. Data from glucocorticoid-induced osteoporotic rats demonstrated for the first time that tetramethylpyrazine (TMP), an extract from a traditional Chinese herb with antiapoptotic properties, could protect MSCs from exposure to glucocorticoids by promoting autophagy and might be an effective agent for the prevention and/or treatment of induced osteoporosis due to the use of glucocorticoids [117]. Recently, another study, using local delivery of TMP in bone marrow of aging mice, demonstrated that TMP could eliminate the senescent phenotype of bone marrow MSCs and could be a potential treatment to ameliorate human age-related skeletal diseases [118]. However, corticosteroids are used for a large number of inflammatory conditions and the corticosteroid-induced osteoporosis represents an important problem from their use.

Current knowledge shows that osteoporosis is a multifactorial process. Recently, microRNAs and long non-coding RNA were reported to be implicated in the pathogenetic mechanisms of skeletal disorders, including osteoporosis [119–121]. Osteoclasts were found to regulate the function of osteoblasts via the secretion of microRNA-containing exosomes that selectively inhibit osteoblast activity [12]. let-7, a miRNA family, was found markedly to promote in vitro the osteogenetic and to suppress the adipogenetic capacities of MSCs, and the let-7/HMGA2 axis has a pivotal role in the maintenance of the balance between the MSC-orchestrated osteogenesis and adipogenesis [122], while miRNA-214-5p/TGF-*β*/Smad2 signaling promotes adipogenic differentiation of bone marrow MSCs and might be a new therapeutic target in postmenopausal osteoporosis [123]. Recently, long noncoding RNA named HOXA-AS2 was found to regulate osteogenesis of MSCs via the transcriptional activity of NF-*κ*B and they suggested that HOXA-AS2 might be a new therapeutic target [120]. Another study, using bone marrow MSCs from 3-week-old Sprague Dawley rats, demonstrated that overexpression of the long noncoding RNA X-inactive-specific transcript (XIST), a major effector of the X chromosome inactivation process, significantly inhibited the differentiation of bone marrow MSCs into osteoblasts. In addition, XIST was found significantly upregulated in plasma and monocytes from osteoporotic patients [121]. Teng et al., using an estrogen deficiency-induced osteoporosis mice model, demonstrated a regulatory effect of miRNAs on the multidirectional differentiation ability of MSCs [124]. Another study demonstrated that miR-106b negatively regulated in vitro the osteogenic differentiation of MSCs and that silencing of miR-106b signaling protected mice against glucocorticoid-induced osteoporosis, through promoting bone formation and inhibiting bone resorption [125]. Both studies suggested that microRNAs might be a potential new strategy for treating osteoporosis and bone defects.

Extensive research experience from experimental studies and clinical data indicate a central role for stem cells in osteogenesis and bone tissue remodeling. Pathophysiological disorders and external factors that disturb the normal functioning of MSC differentiation into osteoblasts could lead to the induction of osteoporosis. Thus, stem cells might be proven key players in the search for new therapeutic strategies in the management of osteoporosis.

### 3.2. The Role of Very Small Embryonic-Like Stem Cells to Osteogenesis and Skeletal Regeneration

A decade ago, a population of very small stem cells, with pluripotent properties, was described for the first time as very small embryonic-like stem cells (VSELs). They were first identified in murine adult bone morrow [126] and later in most murine tissues [127]. In humans, VSELs were first isolated from umbilical cord blood [128], and few years later, they were also found in bone marrow and peripheral blood [129]. VSELS are very small in size (3-6 microns) and express the chemokine receptor CXCR4, other markers that are characteristic for embryonic, epiblast, and primordial germ cells, while they do not express characteristics of the haematopoietic lineage [130]. It has been suggested that VSELs are stored during embryonic development in the bone marrow and they work as a deposit of PSCs. They play a crucial role in postnatal tissue regeneration, giving cells for both hematopoietic and nonhematopoietic tissues and renewing the cell populations of all three germ layers. It has been postulated that VSELs are stored during the organogenesis in organs and tissues as pluripotent SCs that during the postnatal life give rise to oligopotent or omnipotent SCs to support tissue turnover and regeneration. It has also been suggested that this pluripotent stem cell population decreases with age [131]. Therefore, VSELs appear to be significantly involved in the renewal of cellular elements in adult tissues and in the repair of damaged organs, while the reduction in their number with age may be associated with aging.

Given these characteristics, VSELs have gain attention on their ability to generate new tissues, both in in vitro and in experimental models. They have demonstrated that human and murine VSELs could express markers of the osteoblastic phenotype and to differentiate into cells with the capacity to generate skeletal structures and to participate in skeletal repair [132, 133]. Havens et al. have shown that VSELs were capable of differentiating into cells that expressed osteocalcin, which is involved in the mineralization of the bone tissue, and serum circulating osteocalcin reflected the level of bone formation, that is indicative of osteoblastic differentiation. Injected VSELs were localized to the murine bone marrow space adjacent to bony trabeculae and produced robust mineralized tissue by 3 months [133, 134]. The development of mineralized bone tissue and the achieved bone volume were found to be dependent on the number of the injected cells [132]. VSELs seem to be promising for use in regenerative medicine, as autologous treatment of skeletal disorders and to decelerate the aging processes in bone metabolism that leads to osteoporosis.

Several studies have focused on the use of several pharmaceutical agents to recruit endogenous SCs to the osteoporotic tissues, instead of transplanting exogenous stem cells. As we mentioned above, PTH and parathyroid hormone-related protein (PTHrP) are anabolic agents and their administration at low doses and in an intermittent manner induce bone remodeling. It is worth to notice that MSCs have been found to express the PTH receptor on their membranes [135]. These data may help to explain an old observation that PTH exerts its anabolic action, among other mechanisms, stimulating, the first week after its administration, the proliferation and differentiation of MSCs into osteoblasts [136]. In addition, PTH recruits endogenous bone marrow MSCs and induces their adhesion on the trabecular bone surface, starting within 2 weeks from the time of its administration [137]. Furthermore, we have recently observed in our laboratory that a population of VSELs, isolated from peripheral blood and bone marrow of healthy donors, express the PTH receptor (unpublished data). Taken together the physiological role of SCs in bone regeneration and the effects of PTH receptor analogues on osteogenesis, these cells might lead to better and more efficacious cell-based treatment for osteoporosis. A plausible therapeutic option worth investigating would be the use stem cells from young healthy donors with intermittent PTH receptor analogues, such as PTH and PTHrP.

### 3.3. Stem Cell Therapy: Learning from Animal Models of Osteoporosis

Experimental animal models are key tools for the development of new drugs and therapies as they provide important information on the preclinical assessment of their efficacy and safety. Despite the important information provided by animal models in preclinical studies, there are significant differences between the pathophysiological and immune disorders of experimental animals and human diseases. These differences must be carefully taken into account by researchers who would like to translate findings from preclinical studies into therapeutic strategies, mainly when developing biologics or cell therapies [138]. Stem cell transplantation and cell-based therapies have been suggested, and several studies, using experimental animal models, have been conducted for the treatment of the common bone and joint diseases, including osteoporosis [25, 101]. Given that preclinical studies are essential for organizing future clinical trials [139], it is very important to use experimental animal models for osteoporosis to check for safety and to prove the effectiveness of stem cell-based therapy, taking into account the stem cell origin, the route of administration for transplant, and the osteoporotic animal model that has been used (Table 1).

The majority of experimental animal studies were conducted in small animal models such as mice [140, 141] and rats [142], while only a small number of studies used rabbits [143, 144]. Osteoporosis was induced mostly by ovariectomy [143, 145] or by administration of glucocorticoid [13, 146], while Aggarwal et al. developed an immune-deficient osteoporotic murine model [141]. Among these osteoporosis animal models, the rat ovariectomy model seems to be the most preferred animal, with mice to follow, as ovariectomy leads to bone loss without hormonal or drug intervention, mimicking postmenopausal osteoporosis in women. Although there are substantial differences between the rat and human skeleton, these could be overcome with the extensive knowledge of the rat skeleton and various experimental techniques as body mineral density (BMD) is mainly used to prove the effectiveness of the stem cell-based treatment [147]. For studies into the treatment of osteoporotic animals, in addition of embryonic mesenchymal stem cells (ESCs), stem cells of various origin have mostly been used, such as mesenchymal stem cells derived from adipose tissue (ADSCs), from bone marrow (BM-MSCs), and from umbilical cord blood (UCB-MSCs).

MSCs were found to exert significant therapeutic potential through differentiating into various musculoskeletal tissues and thereby mediating tissue repair or by secreting a plethora of soluble mediators, including growth factors and immunomodulatory cytokines [148]. These properties have inspired researchers to investigate the effect of transplanting MSCs locally (to the bone surface) and systemically in osteoporotic animal models [149, 150]. However, MSCs do not have the capability of spontaneous engraft and differentiation depending on the therapeutic target and the relevant target areas. Thus, genetically modified MSCs were used to increase engraftment and differentiation at osteoporotic bone areas [13, 151]. In an ovariectomy (OVX) mouse model, bone-marrow derived MSC's were observed to be home to bone, after 2 months of intravenous injection leading to increased BMD and restored bone volume [152]. Adipose-derived MSCs injected intravenously in OVX mice induced an increase of BMD at 4 and 8 weeks post injection [153]. Transplantation of adipose-derived MSCs, infected with lentiviral vectors expressing alpha-1 antitrypsin, was found to reduce bone loss in OVX mice. Interestingly, the migration of the transplanted cells into the bone tissue resulted to alpha-1 antitrypsin secretion, a significant decrease of the serum IL-6 and IL-1*β* cytokines, and a reduction in the gene expression of RANK in the bone [154]. Intravenous transplantation in OVX osteoporotic mice of MSCs that overexpress, after retroviral transduction, the SDF-1 receptor CXCR4 or the RANK-Fc, an antagonist for RANK-L, was found to increase the cell trafficking into the bone tissue. This procedure protected against bone loss and enhanced the positive effect of RANK-Fc in osteoporosis treatment [155]. Another similar study, using a senescence-accelerated mouse model that expresses a phenotype of progressive aging and injecting through the tail vein MSCs or conditioned media from MSC, demonstrated a decrease in osteoporosis progression as a result of both maintaining osteocalcin production and inhibiting bone marrow adipose tissue accumulation [156]. These data suggest that MSC transplantation may prevent or treat human age-related osteoporosis providing a model for developing cell-based therapies using MSCs. There is evidence of a substantial decrease of the proliferation rate and of the ability to differentiate in aged MSCs. This is additionally supported by data from experimental models where MSCs from bone marrow of young and old rats transplanted in osteoporotic mice were compared. Transplantation of young MSCs to osteoporotic mice resulted in significantly higher BMD compared to the administration of aged MSCs [157]. This raises the question are MSCs from a younger source more plausible to be used for therapy than autologous older cells or cells that have been expanded (as expansion ages cells)?

A number of experimental studies of stem cell-based therapy in osteoporotic animals have used ADSCs. These cells are more easily isolated, compared to their bone marrow counterparts, and easier to produce a larger number of isolated stem cells for transplantation [158]. ADSC transplantation in osteoporotic rats and mice significantly increased BMD [159], while the transplantation of young ADSCs showed a statistically significant improvement in bone regeneration, compared to that in mice receiving aged transplants, with a BMD improvement of 24.3% on average [160]. In one of the few studies of stem cell therapies conducted in larger animals, ADSCs transplanted in OVX osteoporotic rabbits were found to enhance bone regeneration and osteogenesis in vivo and to stimulate in vitro proliferation of bone marrow MSCs, followed by osteogenic differentiation [143]. These data combined to similar results from MSC studies, mentioned above, suggest that in stem cell therapy, stem cells isolated from young donors might be more effective in the treatment of osteoporosis.

UCB-MSCs are another rich source of stem cells that hold distinct inherent characteristics making them a first choice for the development of stem cell-based treatments for musculoskeletal disorders [161]. Human UCB-derived CD34(+) cells that were expanded using a nanofiber-based culturing system systematically were transplanted to osteoporotic mice, and they achieved to increase bone deposition and BMD and to improve bone microarchitecture [146]. Four and eight weeks after systemic application of UCB-derived MSCs or conditioned media from their cultures into OVX, osteoporotic mice showed an increase in BMD levels. In addition to UCB-MSCs, other fetal adnexa, such as placenta and amniotic fluid, are an innovative source of nonembryonic SCs, probably suitable for cell-based therapies, mainly in perinatal disorders. MSCs derived from fetal adnexa seem to have higher proliferative potential and multilineage differentiation capacity compared to adult tissue sources [162, 163]. These cells share similar properties with adult MSCs, but *in vitro*, they have a higher expansion rate and differentiation capacity, compared to adult MSCs that could give a number of cells of the adipose and skeletal tissues [162, 163]. Amnion and chorion, placenta-derived structures, are important sources of MSCs, but the expansion ability of these SCs is dependent on the gestational age [164]. Amniotic fluid-derived stem cells, deriving from dermal fibroblast and fibrous connective tissues, express similar properties and embryonic and adult SC markers with MSCs derived from other tissue sources [165]. MSCs derived from chorion differentiate more easily into osteocytes [162], while all the types of fetal adnexa-derived MSCs differentiated into osteogenic, myogenic, chondrogenic, and adipogenic lineages [166]. Another study demonstrated amelioration in bone fractures, using intrauterine human embryonic MSC transplants isolated from blood of the first trimester donors, in osteogenesis imperfecta mice [167]. Finally, mesenchymal stem cells, derived from fetal adnexa, are less implicated in ethical issues than embryonic stem cells, as these tissues are discarded after delivery [167].

There is very little evidence in the literature, and it is under investigation, whether MSCs differentiate into their target tissue, even when desired clinical effects are observed. In most studies, implantation of MSCs in the bone was not detected and it has been suggested that the improvement of the bone loss is a result of a secretory paracrine mechanism instead of direct differentiation into target tissue [145]. In fact, nearly all evidence suggests that the mechanism of regeneration by MSCs is via a paracrine effect on the multitude of growth factors and immunomodulatory cytokines and microparticles, such as exosomes, that they secrete [168]. A recent study, using green fluorescent protein-labeled bone marrow MSCs demonstrated that donor MSCs homed and inhabited, for at least 4 weeks, recipient bone marrow and prevented recipient bone marrow cell apoptosis, while donor MSCs committed to Osterix+ osteoblast progenitors and induced osteoblastogenesis [169].

Despite that several studies have successfully directed iPSCs cells in specific cell lineages, the results from attempts to specifically induce osteogenic differentiation of iPSC cells are poor and limited so far. A number of studies have demonstrated differentiation of iPSCs into osteoblasts, using similar protocols that induced differentiation of embryonic SCs and MSCs into osteoblasts [170–172]. Similarly, scaffolds were found to increase differentiation potential of iPSCs into osteoblasts with less problems compared to MSC cultures [173, 174]. The potential functional benefit of iPSC-derived bone tissue for cell replacement therapy has been confirmed in animal models. Human iPSCs, implanted on long bone segmental defects in rats, demonstrated an osteogenic differentiation and an in vivo osteogenic potential [175]. Implantation in mice of iPSC-derived osteoblasts, after a 12-week period, resulted in differentiation of iPSCs into mesenchymal lineages of the bone, cartilage, and fat [176]. Another study, using a coculture of human-induced pluripotent stem cell-derived mesenchymal stem cells (hiPSC-MSCs) with human umbilical vein endothelial cells implanted on in a cranial bone defect model in nude rats demonstrated an increased (46.38%±3.8%) new bone generation [177]. These characteristics suggest that iPSCs have the potential to be used in future bone regenerative therapies, provided that they would meet the clinical safety standards [178].

Existing data from experimental studies have shown that the use of SCs is very promising for developing cell-based therapies for the treatment and/or probably the prevention of osteoporosis. Recently, a meta-analysis that examines 12 animal model studies of osteoporosis indicates that treatments based on SC therapies significantly improved the BMD [159]. This meta-analysis includes studies that used SCs of various origins, and the SCs were mainly transplanted by intrabone marrow injection and intratail venous injection. Analysis of the specific characteristics of this study revealed that the SC therapies significantly improved the BMD, regardless of the route of transplantation, the method of osteoporosis induction, and the type and amount of used stem cells, with the only exception of the transplantation of embryonic SCs that did not significantly improve the BMD of the osteoporotic animals.

Studies from animal models so far have yielded important knowledge on the methodology of using SCs in the treatment of osteoporosis and strong evidence suggesting a positive future contribution of SC therapy to the severe global medical issue of osteoporosis [179]. In addition, we have learned many mechanisms underlying the significant improvement of the BMD in osteoporosis after SC-based treatments. Current knowledge indicates two possible beneficial effects of stem cells. On one hand, transplanted SCs migrate to the site of injury regardless the route and they are differentiated to assist in tissue repair via their regenerative properties. On the other hand, migrating SCs, producing immunomodulatory cytokines and growth factors, affect the local environment and “renovate” the niche, helping local cells to recover and inducing the recruitment of new cells in the area [96]. Transplantation of UCB-MSCs in collagen-induced arthritis mice with osteoporosis has been found to improve impaired osteogenic differentiation ability via the inhibition of TNF-*α* [180]. More knowledge from basic research and in vivo experiments must be acquired to further understand the pathophysiology of bone regeneration and the integration of the underlying mechanisms of SC involvement in this process. However, the existing animal model data from the preclinical studies along with some early clinical information of SC transplantation provide a pathway for organizing specific clinical trials and using SCs in the treatment and the management osteoporosis.

### 3.4. Stem Cell Therapy: Clinical Trials

Results from experimental animal models and sporadic clinical reports suggest that the use of SCs and regenerative medicine could provide a potential new therapeutic strategy in the management of osteoporosis. However, although this idea has recently received a considerable attention, clinical trials of stem cell transplantation in osteoporotic humans have not been published so far. In humans, the available information mainly comes from studies that transplanted intravenously MSCs for the treatment of osteogenesis imperfecta in children. Transplantation of MSCs seems to improve the bone structure and stability, growth, and fracture healing, but there is limited experience in this area so far [181]. A clinical study of allogeneic MSC transplantation in six children with osteogenesis imperfecta demonstrated that MSCs were safely administered and differentiated to osteoblasts, capable of extending the clinical benefits of bone marrow transplantation, with five out of six patients showing improvement during the first 6 mo post infusion [182]. Another study reported the cases of prenatal human fetal transplantation in two patients with osteogenesis imperfecta, who also received a postnatal boosting with the same cell population. They demonstrated that prenatal transplantation of allogeneic MSCs appears to be safe and of clinical benefit and that a retransplantation is feasible [183]. Taketani et al. administered allogeneic MSCs, ex vivo expanded, in two patients with severe hypophosphatasia, performing multiple MSC infusions. Both patients had been transplanted with bone marrow, received from asymptomatic relatives. They demonstrated that multiple MSC infusions were effective with clinical improvement of the patients with lethal hypophosphatasia and without adverse events [184]. These clinical studies of MSC transplantation for the treatment of osteogenetic disorders present promising clinical results for the use of stem cell therapies in osteoporosis. However, the limited information to date means that it is not possible to be conclusive and that further experimental and clinical studies are required. Currently, only two clinical trials in phase I/II are being conducted that are related to the use of SCs in the management of osteoporosis, as assessed in https://www.clinicaltrials.gov.

Contemplating the use of SCs in the development of cell-based therapies for osteoporosis, two main questions arise. First, which source of SCs is the most appropriate to be used, and second, which administration route would better drive stem cells in the target tissue? A large recent meta-analysis of animal model studies of osteoporosis demonstrated that transplantation of SCs significantly improved osteoporosis regardless of the administration route that they used [159]. Implantation of MSCs, derived from adipose tissue, bone marrow, or umbilical cord blood, was found to significantly increase the BMD compared to controls; in contrast, the use of transplants from embryonic SCs did not induce BMD improvement [159]. Ideally, MSCs should be administrated via systemic transplantation in order to allow them to reach the osteoporotic tissue and treat osteoporosis. There are currently three main sources of MSCs, placental cord blood, bone marrow, and adipose tissue that have been used for research into stem cell therapies. Although very little research has yet to be performed in humans, these sources of MSCs have been used clinically in trials in humans for exploring effectiveness in other diseases [158, 185]. ADSCs have received particular attention in recent years because these cells are easier to be isolated and in large quantities compared to bone marrow MSCs and their inherent characteristics and safety make them attractive for cell-based therapies [157, 160, 186]. This has led many researchers to use ADSCs in bone research, such as in the treatment of osteoporosis. The use of ADSCs is limited when it comes to individuals with very low percentage of body fat and complexities of repeat lipoaspirations when further treatments are required. In these cases, MSCs derived from umbilical cord blood are an important source of SCs in the cell-based therapies. UCB-MSCs have particular characteristics that make them a distinct source of stem cells. First, it is easy to be isolated without requiring any risk procedure for the donor; second, they have strong differentiation capacity and low immunogenic potential; and third, they are from a youthful source [187–189]. Although, these characteristics make UCB-MSCs a distinct source of SCs for cell-based treatment of osteoporosis, we have to take into attention the results from experimental animals that demonstrated a limited effect of embryonic stem cell transplantation in osteoporotic animals [159]. On the other hand, due to the safety and ethical issues raised by the use of induced pluripotent stem cells (iPSCs) and embryonic SCs, many researchers use MSCs in clinical studies of cell therapies [178]. Furthermore, since MSCs could be easily received from several tissues, as mentioned above, and they are multipotent adult cells, over the past decade, more and more clinical studies have been using MSCs. In addition, the therapeutic effect of MSCs has been examined in immune disorders due to their ability for immunosuppression [190]. Possible clinical applications for MSCs in the musculoskeletal system have mainly focused on acute or chronic disorders, such as bone fractures, osteoporosis, cartilage lesions, or ligament injuries [178]. Finally, as mentioned above, the new population of VSELs may hold promise in regenerative medicine and cell-based therapies and it would be possibly efficient to use these cells in clinical studies for treating osteoporosis, because they could be used as an autologous transplantation from peripheral blood.

Data from basic research studies as well as from the experimental animal models show encouraging results for the use of SCs for the development of cell-based therapies for osteoporosis. However, before proceeding with clinical studies on the development of these therapeutic approaches for osteoporosis or other degenerative diseases, several factors should be taken into account and a number of issues should be examined and addressed regarding the isolation and the retention of their reparative capacity [93, 191]. The safe and effective use of MSC should calculate the optimal number of cells to be transported in order to achieve the best clinical outcome in patients with osteoporosis without any side effect or lack of efficacy. If large numbers are required (as expected), MSCs would need an in vitro expansion with a cocktail of growth factors and cytokines. These protocols have been well characterized and can produce high quality of cultures that could produce large numbers of cells for the clinical studies. However, these techniques, used for MSC culture and expansion, have also been shown to lead to an alteration in their reparative capacity [192, 193]. Furthermore, long-term cold storage of MSCs and how that affects the function of MSCs will need to also be examined, if “banking” of MSCs is required [194, 195]. Another thing to consider, from an autologous perspective, is the age of the patient. The older the patient, the more likely that the isolated MSCs will be less in number and less in reparative function due to the loss of lineage specificity, depletion due to self-renewal over the years, and depletion due to senescence that has been seen with age [191, 196, 197]. This suggests that from an autologous approach, the patients should be at a coherent age when doing trials. These are complex issues, and probably, it will take a long experimental and clinical research time to resolve, before we establish new therapeutic strategies, using stem cells, obtained from various sources, like adipose tissue, embryonic vessels, VSELs, and iPSCs, for the treatment of damaged tissues, including osteoporosis.

## 4. Conclusions

Osteoporosis is a common disease in both the developing and developed countries that primarily affects elderly people. Current treatments attempt to target the imbalance in the osteoblast-osteoclast axis. Although these treatments have demonstrated some benefits, limited efficacy and adverse effects complicate them. The development of new strategies is rapidly becoming a dire necessity to meet the rising human and economic toll of osteoporosis. Despite the multifactorial nature of this disorder, stem cells maintain a central role in the pathogenetic mechanisms as their differentiation into osteoblasts promotes osteogenesis and bone remodeling. As osteoporosis is related to a decline in the number and function of osteoblasts, substantial evidence indicates that stem cell stimulation and transplantation have the ability to reverse bone demineralization. Current knowledge supports the concept of using stem cell therapy in osteoporosis, providing evidence of its potential as a new and promising type of treatment with unique advantages.

## Figures and Tables

**Table 1:** Stem cell transplantation in the cell-based therapy of osteoporosis.

Origin of stem cells	Route of administration
Bone marrow, BM-MSCs, and VSELs Umbilical cord blood, UCB-MSCs Adipose tissue, ADSCs Peripheral blood, VSELs Embryonic stem cells (ESCs) Induced pluripotent stem cells (iPSCs)	Intravenous injection Intrabone marrow injection Injection to bone surface Intratail venous injection Intracardiac ventricular injection

MSCs: mesenchymal stem cells; ADSCs: adipose-derived mesenchymal stem cells; VSELs: very small embryonic-like stem cells.
